# Immediate efficacy and persistent speed of kill of a novel oral formulation of afoxolaner (NexGard^TM^) against induced infestations with *Ixodes ricinus* ticks

**DOI:** 10.1186/1756-3305-7-452

**Published:** 2014-09-26

**Authors:** Lénaïg Halos, Wilfried Lebon, Karine Chalvet-Monfray, Diane Larsen, Frederic Beugnet

**Affiliations:** Merial S.A.S., Sanofi Pasteur confluence, 69007 Lyon, France; INRA, UR346 d ’ Epidémiologie Animale, Université de Lyon, VetAgro Sup Campus Vétérinaire de Lyon, 1 Avenue Bourgelat, 69280 Marcy-l’Étoile, France; Merial Limited, 3239 Satellite Blvd, Duluth, GA 30096 USA

**Keywords:** *Ixodes ricinus*, Tick, Afoxolaner, Dog, Speed of kill, Immediate efficacy, Chewable treatment

## Abstract

**Background:**

Ticks are hematophageous arthropods that transmit a wide spectrum of pathogens to human and animals. The ability of an acaricidal product to kill ticks quickly provides an important added benefit, especially as protecting dogs from tick bites remains the best preventive measure against tick-borne diseases. The speed of kill of afoxolaner in a novel soft chewable formulation (NexGard^TM^) against induced infestations with *Ixodes ricinus* adult ticks was evaluated during a full-month negative controlled and blinded study following a single oral administration.

**Methods:**

12 healthy beagle dogs were included and randomly allocated to 2 groups of six dogs each. One Group was a negative control while the other group was treated with an oral formulation of afoxolaner on Day 0. Tick infestations with 40 (±5) female and 10 male adult unfed *I. ricinus* were performed on Days -1, 7, 14, 21 and 28. To evaluate immediate efficacy, the number of live ticks were thumb counted at 12 and 24 hours post treatment. To evaluate the persistent speed of kill following further infestations, ticks were thumb counted 12 and 24 hours post infestations. Ticks were removed 24 hours post treatment or infestation.

**Results:**

Afoxolaner starts to kill the pre-existing tick infestations rapidly with an immediate efficacy of 93.4% and 100% respectively at 12 h and 24 h post treatment. The persistent speed of kill of afoxolaner was significant (p < 0,05), as compared with untreated controls, at 12 hours after infestations at D7 and D21. Efficacy at 12 h was 76.6%, 41.9%, 36.9% and 38.5% at D7, D14, D21 and D28 respectively. Efficacy at 24 h ranged from 91% to 100% for the entire month.

**Conclusions:**

This study demonstrated that besides the excellent acaricidal efficacy of afoxolaner after single oral administration, the product has a rapid speed of kill against one of the most important European tick species and controlled the weekly re-infestations for 28 days post treatment.

## Background

Ticks are major ectoparasites of humans and animals. In addition to their direct pathogenic role, they are also known as vectors for a wide range of pathogenic microorganisms. *Ixodes ricinus* is the most common tick species occurring in Europe. It is the vector of various vector borne pathogens including *Borrelia burgdorferi* (the agent of Lyme disease), *Anaplasma phagocytophilum* (the agent of granulocytic ehrlichiosis) or the virus responsible of tick-borne Encephalitis [1, 2]. The geographic distribution of the tick species is expanding in Europe [3] as a result of climate changes or increasing movements of people travelling abroad with their pets [4] and it represents a major threat for human and animal health. As an example, a recent study demonstrated that almost 60% of the ticks collected from humans in Italy were *I. ricinus* ticks and that 21% of them were carrying at least one pathogenic microorganism [5].

Canine tick-borne diseases are among the most prevalent infectious diseases in the dog population in Europe [6]. The main pathogens transmitted to dogs by *I. ricinus* ticks are bacteria (*B. burgorferi* sensu lato, *A. phagocytophilum*) but a few cases of *Ixodes*-borne viral diseases (TBE-virus, Louping-ill virus) have also been described in dogs in Europe [1]. Protecting dogs from tick bites remains a primary preventive measure against those diseases. Numerous acaricides have been developed for the protection of dogs against ticks which include topical solutions as spray, collars, or spot-on and, more recently, oral formulations. Due to delayed transmission of many tick-borne pathogens [7], a rapid onset of action of an acaricide is likely to have an impact on pathogen transmission [8] and this feature brings an important added value to any acaricidal solution. According to the standard determined by pharmaceutical regulation worldwide, an anti-tick product for dogs should give a minimal activity of 90% acaricidal efficacy, characterized by a reduction in tick counts 48 h after treatment and the prevention of re-infestation [9]. Faster acting acaricidal activity, i.e. within the first hours post treatment or subsequent infestation, is interesting to evaluate, especially from a perspective of reduction of the risk of diseases transmission.

Orally administrated afoxolaner has demonstrated a persistent efficacy against the main species of ticks infesting dogs with >90% efficacy 48 hours post treatment or infestation for one month after treatment against *I. ricinus, I. scapularis, Dermacentor reticulatus, D. variabilis, Rhipicephalus sanguineus, Haemaphysalis longicornis* [10–14].

The objective of the present study was to assess immediate efficacy of this novel oral formulation of afoxolaner (NexGard^TM^, Merial) against an existing *I. ricinus* tick infestation and its persistent preventive speed of kill against subsequent infestations with *I. ricinus* ticks within the first hours post- treatment or subsequent re-infestation.

## Methods

The study was conducted using a negative controlled and blinded design, with dogs randomly allocated to two groups of 6 dogs (treated and control). Prior to treatment, all dogs underwent a physical examination conducted by a veterinarian and daily health observations by trained personnel to ensure that they were healthy. To detect the presence or absence of any treatment-related or unrelated health abnormality or adverse event, health observations were conducted at hourly intervals for four hours after treatment and daily thereafter throughout all studies.

### Study design

The study was designed in accordance with the *World Association for the Advancement of Veterinary Parasitology (W.A.A.V.P.) guidelines for evaluating the efficacy of parasiticides for the treatment, prevention and control of flea and tick infestation on dogs and cats* [15]. All animals were managed similarly, with due regard for their well-being and in compliance with Merial Ethics Committee, other local applicable regulations and requirements (reference numbers of the authorization: APS 10-09-0005 and 1423). The study was conducted in accordance with the International Cooperation on Harmonization of Technical Requirements for Registration of Veterinary Medicinal Products (VICH) [16].

### Animals

Fourteen Beagle dogs were allocated based on pretreatment tick infestation. The 2 dogs with the lowest live attached tick counts were not allocated and were removed from the study. Dogs were older than 4 months, and body weight ranked from 6.6 kg to 10.7 kg on Day -2. They had not been treated with ectoparasiticides (either topical or systemic) within 3 months before the start of the study.

### Ticks

Ticks were from a German laboratory-maintained population that had been established from *I. ricinus* ticks collected in the field in Europe. These ticks were from a strain not known to be resistant to any ectoparasiticide.

### Treatment

On Day 0, dogs from Group 2 were treated with NexGard^TM^ chewable at the commercial dose. Dogs from Group 1 were not treated. Chewable tablets were manually administered into the back of the mouth. No product was rejected during the treatment administration. Personnel involved with evaluation of efficacy were blinded as to treatment.

### Tick infestations and counts

On Days -1, 7, 14, 21 and 28, all dogs were infested with 40 (±5) females and 10 males adult unfed *I. ricinus.*

On Day 0, the immediate efficacy was evaluated by thumb-counting ticks on dogs 12 hours post-treatment. Final count and removal of ticks was performed 24 hours post-treatment.

On Days 7, 14, 21 and 28, ticks were thumb counted 12 hours post-infestation and were counted and removed 24 hours post-infestation for the evaluation of the persistent speed of kill. An infestation rate of >25% in control group was considered to be an adequate infestation.

These counts were performed by methodical examination of the whole body, parting and feeling through the dog’s hair with the finger tips, with further visual confirmation of the tick’s presence. Ticks were categorised as free or attached, live or dead, as described by Marchiondo *et al*. [15].

### Data analysis

The live female tick counts (attached or free) were transformed to the natural logarithm of (count + 1), for calculation of geometric means by treatment group at each time point. The treatment group was listed as the fixed effect and blocks were listed as the random effect. Percent efficacy of each treated group with respect to the control group was calculated using the formula: [(C - T) / C] × 100 where C = geometric mean for the control group and T = geometric mean for the respective treated group. The log-counts of the treated group were compared to the log-counts of the untreated control group using a non-parametric test, the Wilcoxon rank sum test, at each time for the live ticks. The testing was two-sided and used a significance level of 5%. All analyses were performed using the R language version 2.15.3 [17].

## Results

Treated dogs did not show any adverse effect related to treatment.

The geometric mean number of live female ticks ranged between 14.5 and 24.4 for dogs in the control Group, corresponding to a minimum retention rate of 37%, sufficient to make a valid assessment of tick efficacy [15].

The immediate efficacy of afoxolaner against existing tick infestations reached 93.4% within 12 hours after treatment and was complete (100%) 24 hours post-treatment (Table 1).

**Table 1:** **Geometric means of live female tick counts at the scheduled hours following treatment and further induced infestations**

Days of tick count	Day 0		Day 7		Day 14		Day 21		Day 28	
Hours of tick counts^1^	12 h	24 h	12 h	24 h	12 h	24 h	12 h	24 h	12 h	24 h
Number of ticks^2^	14.5	16.4	20.8	24.4	22.4	21.8	19.1	21.2	19.9	19.3
Untreated Control Group										
Number of ticks^2^	1.0	0.0	4.9	0.0	13.0	0.1	12.1	0.7	12.2	1.7
Treated Group (NexGard^TM^)										
**% Efficacy** ^**3**^	93.4**	100.0**	76.6*	100.0**	**41.9**	99.4**	36.9*	96.6*	**38.5**	91.2**

^1^Hours of tick counts are indicated either post-treatment on D0 or post-infestation on days 7, 14, 21, 28.

^2^Geometric means tick counts.

^3^Based on Geometric means.

Treated Group differed statistically significantly from the untreated control Group with:

*(p < 0.05).

**(p < 0.01).

The persistent speed of kill showed a significant reduction in tick counts 12 hours post-infestation when compared to the controls at D7 and D21 (p < 0.05), with efficacy of 76.6%, 41.9%, 36.9% and 38.5% respectively at D7, D14, D21 and D28. The efficacy at 24 hours post-infestation ranged from 91.2 to 100% for each challenge throughout the whole month.

## Discussion

When orally administered, afoxolaner is systematically distributed in the body of the treated dog and plasma concentrations peak quickly within 2 to 6 hours [18]. The immediate efficacy against existing tick infestation is therefore rapid and this is illustrated by the onset of action on experimental infestation with *I. ricinus* ticks which reached >93.4% efficacy 12 hours post-treatment and 100% 24 hours post-treatment.

Attachment and ingestion of material containing afoxolaner is likely required to affect ticks applied after treatment. Although ticks typically start to effectively take blood only 24 hours post-attachment, with the first hours being dedicated to the attachment process [19], fluid exchanges between the tick and the host still occur during the very first phase of attachment, as ticks will ingest a limited quantity of plasma during this phase [20]. The present study suggests that the quantity of afoxolaner in contact with the tick during this fluid exchange is enough to initiate the killing of the ticks. The onset of action is observed within 12 hours and the efficacy is completed at 24 hours post-infestation for the whole month following NexGard^TM^ administration.

These results corroborate the hypothesis that afoxolaner rapidly kills ticks as suggested in a recent study that demonstrated the ability of orally administrated afoxolaner to block the transmission of *Babesia canis* by *Dermacentor reticulatus* ticks to dogs with 100% of protection of the treated dogs [21]. The level of protection against disease transmission depends on the time required for the transmission of the responsible pathogen: it is probably lower against pathogens in which transmission starts earlier, as soon as salivation begins, within a few minutes or hours of attachment such as it has been demonstrated for viruses [22] or suggested for *Rickettsia conorii* [23], but is expected to be high against pathogens requiring more than 24 hours of attachment to be transmitted such as *Borrelia burgdorferi* [24] or *Anaplasma phagocytophilum* [25].

Dynamics and pattern of seasonal activity of ticks have been shown to be changing dramatically in Europe due to both climate changes and non-climatic factors [3]. The risk of *I. ricinus* tick infestation and tick-borne disease transmission is not limited to Spring and Autumn [26]. It can even be very high in winter in some areas of Southern Europe [27]. For an optimal protection of the dogs, the period of treatment has to be adapted to each epidemiological situation.

## Conclusion

In addition to its demonstrated acaricidal efficacy, afoxolaner, administrated orally in NexGard^TM^ formulation, has a quick onset of action against *I. ricinus,* allowing a rapid elimination of the existing infestations and preventing the establishment of further infestations for a month.
